# Hypoxia: a critical pathophysiological driver in respiratory inflammatory diseases

**DOI:** 10.1016/j.mmr.2026.100023

**Published:** 2026-04-18

**Authors:** Bing Zhong, Lan Yang, Si-Lu Sun, Hong Chen, De-Yun Wang

**Affiliations:** a Department of Otolaryngology-Head and Neck Surgery, West China Hospital, Sichuan University, Chengdu 610041, China; b Department of Otolaryngology, Yong Loo Lin School of Medicine, National University of Singapore, 119077, Singapore, Singapore; c Department of Pulmonary and Critical Care Medicine, West China Hospital, State Key Laboratory of Respiratory Health and Multimorbidity, Sichuan University, Chengdu 610041, China; d State Key Laboratory of Oral Diseases, National Clinical Research Center for Oral Diseases, Chinese Academy of Medical Sciences Research Unit of Oral Carcinogenesis and Management, West China Hospital of Stomatology, Sichuan University, Chengdu 610041, China; e Department of Neurology, Shengli Clinical College of Fujian Medical University, Fujian Provincial Hospital, Fuzhou University Affiliated Provincial Hospital, Fuzhou 350001, China

**Keywords:** Respiratory inflammatory diseases, Hypoxia, Hypoxia-inducible factor, Epithelial barrier, Immune imbalance, Inflammation

## Abstract

Hypoxia is a central pathophysiological driver of inflammatory airway diseases, shaping disease progression largely through hypoxia-inducible factor 1α (HIF-1α) signaling. Across these disorders, hypoxia exacerbates airway inflammation through shared mechanisms. As a key signaling hub, HIF-1α disrupts epithelial barrier integrity and initiates inflammatory cascades; reprograms immune responses, promoting the activation and trafficking of eosinophils, T cells, and macrophages while reshaping cytokine profiles, to drive tissue injury; and accelerates airway remodeling, thereby worsening airflow limitation and perpetuating inflammatory cycles. Realizing effective targeted therapies will require rigorous validation of HIF-1α as a therapeutic node and the development of disease-tailored interventions aligned with distinct pathological features. In parallel, strengthened translational and clinical research on hypoxia is essential to build a robust evidence base for practice. This review synthesizes hypoxia-driven mechanisms shared across airway diseases, articulates a unifying framework for HIF-1α signaling across pathological contexts, and highlights the therapeutic implications of fundamental discoveries. By addressing the paucity of cross-disease analyses of hypoxia pathways, it provides both a conceptual foundation and a practical roadmap for developing precise and efficient targeted therapies for inflammatory respiratory diseases.

## 1 Background

Respiratory inflammatory diseases, including chronic rhinosinusitis (CRS) [1], [2], allergic rhinitis (AR) [3], [4], asthma [5], [6], chronic obstructive pulmonary disease (COPD) [7], cystic fibrosis (CF) [8], and respiratory viral infections, share several key epidemiological features [9]. They are highly prevalent worldwide and collectively affect a substantial proportion of the population (CRS: 12%; AR: 400 million; asthma: 262 million; COPD: 10%; CF: 89,000) [7], [10], [11], [12], representing a leading cause of chronic morbidity [13], [14]. Many begin early in life and follow a relapsing and progressive course, with disease strongly influenced by environmental exposures, including air pollution, tobacco smoke, indoor allergens, occupational irritants, and respiratory pathogens [10], [15]. These conditions also frequently co-occur, for example, rhinitis with asthma, and viral infections precipitating COPD or asthma exacerbations, supporting the concept of a shared “united airway” susceptibility [16], [17]. Clinically, severity spans mild, self-limited symptoms to chronic disability, recurrent exacerbations, and life-threatening respiratory failure, making these diseases major contributors to healthcare utilization, productivity loss, and global mortality [18], [19], [20], [21], [22], [23].

**Fig. 1:**
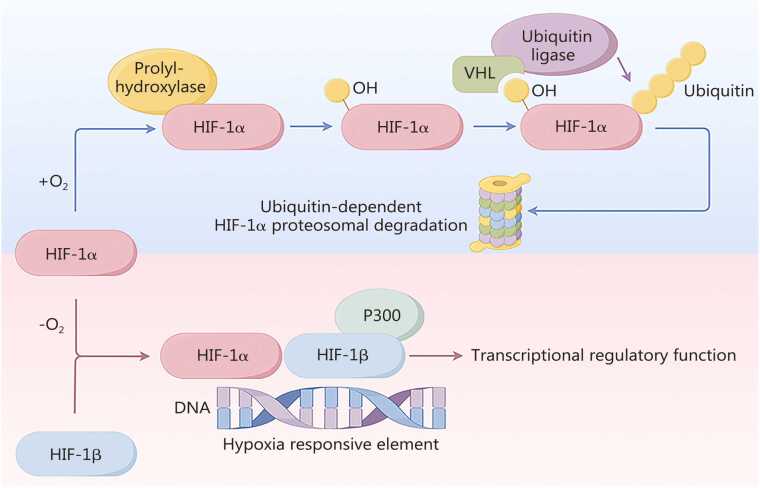
Control mechanisms for HIF in both normoxia and hypoxia. In normal oxygen conditions, HIF-α undergoes hydroxylation through PHD and FIH enzymes, triggering ubiquitination and subsequent degradation mediated by VHL. However, under hypoxic conditions, HIF-1α circumvents degradation by inhibiting PHD/FIH, allowing its transportation into the nucleus. Once inside the nucleus, HIF-α associates with co-activator p300/CBP and forms a complex with HIF-1β, initiating gene transcription. HIF. Hypoxia-inducible factor; PHD. Prolyl hydroxylase domain; FIH. Factor-inhibiting HIF; VHL. Von Hippel-Lindau; p300. E1A-binding protein p300; CBP. CREB-binding protein.

Hypoxia is thought to play important roles in both physiological and pathological states at the organismal and cellular levels [24], [25], [26], [27]. Hypoxia-inducible factors (HIFs) are heterodimeric transcription factors composed of an oxygen-regulated α subunit and a constitutively expressed β subunit [28], [29], [30], [31]. Under normoxic conditions, prolyl hydroxylase domain proteins (PHDs) and the asparaginyl hydroxylase factor inhibiting HIF (FIH) hydroxylate HIF-α [24], [32], promoting its recognition by the von Hippel-Lindau (VHL) E3 ubiquitin ligase complex and subsequent proteasomal degradation [28], [33]. However, under hypoxic conditions, hydroxylation is reduced, preventing VHL-mediated ubiquitination and allowing HIF-α to accumulate, translocate to the nucleus, dimerize with HIF-β, and recruit the co-activators p300/CREB-binding protein (CBP) to activate downstream target gene transcription [29], [32], [34]. Among the three HIF-α isoforms, HIF-1α and HIF-2α are the most extensively studied because of their prominent roles in hypoxia responses [33], [35], [36] **(**Fig. 1**)**.

Recent studies indicate that hypoxia, particularly through HIF-1α activation, acts as a key regulator in the pathogenesis of airway inflammatory diseases [37], [38], [39], [40]. HIF-1α can be induced by diverse stimuli and, when upregulated, may injure the respiratory epithelium, impair barrier integrity, and promote the loss of junctional proteins, including tight-junction components. These changes are associated with increased vascular endothelial growth factor (VEGF) expression and activation of proinflammatory signaling pathways such as phosphoinositide 3-kinase (PI3K)-protein kinase B (Akt), thereby enhancing inflammatory mediator production [41], [42]. Together, these processes contribute to airway inflammation and structural remodeling. Hypoxia also shapes immune responses and can promote immune imbalance. In a study of 23 patients with AR, HIF-1α and aryl hydrocarbon receptor (AhR) expression in CD4⁺ T cells was higher than in controls under both hypoxic and normoxic conditions, supporting a role for HIF-1α in immune-cell activation [43]. Through regulatory pathways including Ras/mitogen-activated protein kinase (MAPK) and sirtuin1 (SIRT1), hypoxia may further influence eosinophil survival, dendritic-cell function, and Th17 responses [44], [45], exacerbating mucosal barrier dysfunction, immune disequilibrium, airway inflammation, and vascular remodeling. This mechanistic link between hypoxia and respiratory disease has motivated efforts to therapeutically modulate HIF-1α activity. Pharmacologic strategies targeting upstream regulators such as peroxisome proliferator-activated receptor-γ (PPAR-γ) and SIRT1 have been reported to suppress HIF-1α and VEGF expression, with potential to reduce inflammation and angiogenesis associated with disease severity [46], [47], [48], [49], [50], [51]. In addition, environmental and lifestyle approaches that reduce exposure to allergens and pollutants may help maintain HIF-1α homeostasis and thereby prevent respiratory symptom exacerbations [52], [53].

Given the central role of hypoxia in respiratory tract inflammation, this review synthesizes evidence across CRS, AR, asthma, COPD, CF, and respiratory viral infections to position hypoxia as a shared, potentially actionable driver and to delineate convergent hypoxia-dependent pathways. We focus on how hypoxia coordinates pathogenic processes across airway compartments and disease phenotypes, and we evaluate both the therapeutic promise and potential risks of pharmacologically modulating the hypoxia-HIF axis. By linking molecular mechanisms to clinical endotypes and unmet treatment needs, we argue that selectively targeting hypoxia-driven HIF-1-signalling may support more precise, stage- and phenotype-informed interventions beyond broad anti-inflammatory strategies, helping advance rational personalized approaches to improve long-term outcomes and reduce the burden of inflammatory respiratory disease.

## 2 Involvement of hypoxia in inflammatory and infectious airway diseases

Hypoxia is a key pathogenic factor for respiratory diseases. Chen *et al*. [54] reported that serum HIF-1α levels correlate with lung function and blood gas parameters in patients with COPD. Consistently, Lee *et al*. [55] found higher HIF-1α and VEGF expression in lung tissue from smokers with COPD than from smokers with preserved lung function. Using genome-wide association studies, Anand *et al*. [56] identified HIF-1α as a key signaling mediator in AR. Together, these clinical data underscore the translational relevance of hypoxia-associated pathways in airway disorders. In an integrative analysis of smokers with asthma, COPD, or asthma-COPD overlap (ACO), Fangal *et al*. [57] further showed that ACO is characterized by distinct physiological and radiological features alongside transcriptomic enrichment of the HIF-1 signaling pathway. A notable feature of hypoxia in these conditions is its capacity to drive both shared pathogenic programs and disease-specific changes [56], [58], [59]. Specifically, hypoxia contributes to epithelial injury and dysfunction (e.g., in asthma, AR, and CF) [60], [61], [62]; induces local immune dysregulation by modulating the differentiation and functional status of specific immune cell subsets [18], [46], [47], [48], [49], [50], [51], [52]; promotes tissue remodeling in asthma and COPD, stimulates airway smooth muscle hyperplasia and excessive contraction, and exacerbates airway hyperresponsiveness and airflow limitation [57], [63], [64], [65], [66], [67], [68], [69]; and mediates the hypoxia-inflammation-viral replication vicious cycle, thereby facilitating respiratory viral infections such as those caused by severe acute respiratory syndrome coronavirus 2 (SARS-CoV-2) and influenza A (H1N1) virus [70], [71]. Notably, immune dysregulation arising from hypoxia, driven shifts in immune cell differentiation and function appear to be a recurrent feature across these airway diseases.

### 2.1 Chronic rhinosinusitis

CRS is a chronic inflammatory disorder of the sinonasal mucosa that is commonly categorized into CRS with nasal polyps (CRSwNP) and CRS without nasal polyps (CRSsNP) [72], [73]. Eosinophilic CRSwNP is typically characterized by type 2 inflammation [74], [75]. Increasing evidence indicates that elevated HIF-1α expression contributes to CRS pathogenesis [76]. In inflamed and obstructed sinuses, impaired ventilation together with mucosal edema lowers local oxygen tension, generating a sustained hypoxic microenvironment. This hypoxia activates HIFs, particularly HIF-1α, which reprogram gene expression in epithelial cells, fibroblasts, and immune cells [61], [77]. Functionally, hypoxia can weaken epithelial barrier integrity, reduce ciliary beat frequency and mucociliary clearance, and promote accumulation of viscous, stagnant mucus, thereby facilitating bacterial colonization and biofilm formation [78], [79]. In parallel, hypoxia enhances the production of proinflammatory cytokines and growth factors that drive tissue remodeling, angiogenesis, and edema, further narrowing sinus ostia and perpetuating impaired aeration [80], [81], [82]. In specific CRS endotypes, especially those marked by prominent non-eosinophilic inflammation with nasal polyps, hypoxia may also potentiate type 1 immune responses and oxidative stress, aggravating symptoms and disease severity [79], [83]. Clinically, these processes establish a feed-forward loop in which inflammation promotes obstruction and hypoxia, while hypoxia sustains and amplifies chronic inflammation [79], [84], [85]. Overall, current findings position hypoxia as a mechanistic link between sinonasal obstruction and persistent inflammation, impaired mucosal defense, and tissue remodeling in CRS. Key next steps include mapping hypoxic niches across CRS endotypes, developing and validating biomarkers that reliably reflect tissue hypoxia and HIF activity, and testing interventions that restore ventilation and mucociliary function or selectively modulate HIF-1α-dependent programmes.

### 2.2 Allergic rhinitis

AR is an IgE-mediated inflammatory disease of the nasal mucosa in which allergen exposure triggers mast cell and basophil activation and the release of mediators such as histamine and leukotrienes [86], [87]. Within this cascade, hypoxia can arise as a downstream consequence of mucosal swelling, vascular leakage, and congestion and can, in turn, amplify AR through activation of hypoxia-responsive signaling pathways [37], [38], [39], [40], [41]. Specifically, nasal obstruction, mucosal edema, and increased vascular permeability reduce oxygen availability in the nasal epithelium and lamina propria, generating a transient or persistent low-oxygen microenvironment. Under these conditions, HIF-1α is stabilized and translocates to the nucleus, where it regulates transcriptional programs linked to cellular metabolism, inflammatory signaling, and vascular responses [41], [58]. Hypoxia may also compromise epithelial barrier function by disrupting tight junctions, impairing mucociliary clearance, and increasing expression of adhesion molecules, thereby facilitating allergen penetration and prolonging the time of allergen stimulation on the mucosa, thereby promoting the activation of immune cells [88], [89].

### 2.3 Asthma

In asthma, allergen exposure in sensitized individuals triggers an IgE-mediated response that activates mast cells and basophils, leading to the release of inflammatory mediators such as histamine and leukotrienes and promoting allergic airway inflammation, bronchoconstriction, and airway hyperresponsiveness [90], [91]. In this context, excessive or recurrent hypoxia can increase HIF-1α expression and contribute to airway inflammation [80], [92], [93], [94]. During acute bronchoconstriction and chronic airway remodeling, airflow limitation produces regional ventilation and reduces oxygen tension in the conducting airways and distal lung [95], [96], [97]. The resulting low-oxygen milieu stabilizes HIF-1α in both structural and immune cells, reprogramming gene expression toward pathways that support inflammation, mucus hypersecretion, and tissue remodeling [41], [98], [99]. Hypoxia also induces VEGF and other angiogenic mediators, promoting vascular remodeling of the airway wall, edema, and thickening that further exacerbate airflow obstruction [41], [100]. In airway smooth muscle, hypoxia may enhance contractility and stimulate proliferation, thereby worsening bronchial hyperresponsiveness and contributing to fixed airflow limitation over time [63], [101]. In parallel, local and systemic hypoxia can increase oxidative stress, compromise epithelial barrier integrity, and potentially modulate glucocorticoid responsiveness, which may influence disease control and treatment outcomes [102], [103]. Clinically, nocturnal and exercise-induced hypoxemia have been linked to more severe asthma, more frequent exacerbations, and worse prognosis [104], [105]. Collectively, these findings indicate that hypoxia is not only a consequence of airflow obstruction in asthma but also a feed-forward driver that amplifies and sustains chronic airway inflammation and remodeling.

### 2.4 Cystic fibrosis

CF is an inherited disorder caused by mutations in the cystic fibrosis transmembrane conductance regulator (CFTR) gene. CFTR dysfunction impairs epithelial ion transport, dehydrates the airway surface liquid, and disrupts mucociliary clearance, leading to mucus retention and chronic infection [12], [106], [107], [108]. These obstructed airway regions develop localized zones of low oxygen tension, particularly within thickened mucus plugs and bacterial biofilms. In this hypoxic microenvironment, pathogens such as *Pseudomonas aeruginosa* undergo metabolic adaptation, form more resilient biofilms, and become less susceptible to antibiotics and host immune defenses [62], [109]. Meanwhile, hypoxia promotes stabilization and accumulation of HIF-1α in airway epithelial and immune cells, reprogramming gene expression toward heightened proinflammatory cytokine production, increased neutrophil recruitment, and persistence of chronic inflammation [110], [111], [112], [113]. The resulting neutrophil-dominated inflammatory response contributes to airway tissue injury and remodeling and further compromises mucociliary clearance, thereby worsening mucus obstruction and sustaining the inflammation cycle that drives CF progression [24], [112], [114], [115], [116].

### 2.5 Chronic obstructive pulmonary disease

COPD is characterized by persistent airway inflammation driven largely by cigarette smoke and environmental pollutants, leading to aberrant tissue repair and airway remodeling that promote emphysema and incompletely reversible airflow limitation [117], [118], [119]. Increasing evidence implicated HIF-1α as an important mediator in COPD pathogenesis [54], [120], [121], [122], [123], [124], [125], [126], [127]. Persistent airflow limitation, small-airway obstruction, and emphysematous alveolar destruction cause ventilation-perfusion mismatch and impaired gas exchange, resulting initially in exertional and nocturnal hypoxemia and, in advanced disease, chronic resting hypoxemia [128], [129]. Reduced oxygen tension stabilizes and activates HIF-1α in pulmonary vascular, epithelial, and inflammatory cells, inducing transcriptional programs that favor vasoconstriction and vascular remodeling, thereby contributing to pulmonary hypertension, increasing right-ventricular afterload, and predisposing patients to cor pulmonale [118], [125], [130]. Within the airways and lung parenchyma, hypoxia can further amplify inflammation, increase oxidative stress, and impair epithelial repair responses, accelerating structural injury and functional decline [131], [132]. During acute exacerbations, episodes of more profound hypoxemia may intensify inflammatory signaling and tissue damage, which can hasten loss of lung function over time [133], [134], [135]. Collectively, these observations support the view that hypoxia in COPD is not only a downstream consequence of chronic airflow obstruction but also a disease-modifying factor that promotes pulmonary vascular pathology, systemic comorbidities, and overall disease severity.

### 2.6 Viral infection in the respiratory tract

Hypoxia is both a consequence and a pathogenic amplifier of respiratory viral infections such as respiratory syncytial virus (RSV) [136], influenza A [137], and SARS-CoV-2 [138], [139]. Viral replication and virus-driven inflammation promote airway edema, mucus plugging, and alveolar capillary injury, causing ventilation-perfusion mismatch and impaired oxygen diffusion [140], [141]. The resulting low oxygen tension stabilizes HIFs in epithelial and immune cells [142], [143], reshaping gene expression toward a more proinflammatory, pro‑thrombotic, and glycolytic phenotype [138], [144], [145]. HIF activation can increase cytokine and chemokine production, promote neutrophil and monocyte recruitment, and modulate antiviral interferon signaling, potentially influencing viral clearance while also raising the risk of immunopathology [146], [147], [148]. In severe influenza and coronavirus disease 2019 ‌(COVID-19) pneumonia, persistent hypoxemia combined with endothelial injury and microvascular thrombosis can further worsen pulmonary and systemic tissue hypoxia, contributing to multiorgan dysfunction.

**Fig. 2:**
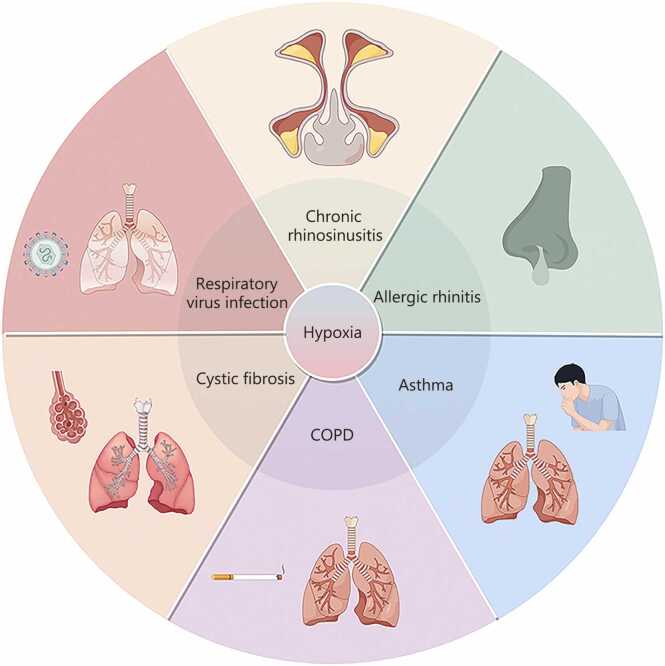
Hypoxia drives the pathogenesis and progression of respiratory diseases. Hypoxia accelerates the onset of upper respiratory tract disorders, including chronic rhinosinusitis and allergic rhinitis, while also facilitating the progression of lower respiratory tract diseases such as asthma, cystic fibrosis, and chronic obstructive pulmonary disease; hypoxia can also promote respiratory virus infections. COPD. Chronic obstructive pulmonary disease.

Taken together, evidence across CRS, AR, asthma, CF, COPD, and viral infections supports the concept that hypoxia is not merely a downstream byproduct of disease but a central driver of pathogenesis **(**Fig. 2**)**. Although disease manifestations differ, barrier dysfunction in asthma and AR, impaired mucociliary clearance in CF, vascular remodeling in COPD, or dysregulated antiviral responses in respiratory infections, a unifying theme is that hypoxia amplifies inflammation and tissue injury through conserved mechanisms, including HIF-1α stabilization, VEGF induction, and immunometabolic reprogramming. At the same time, disease-specific microenvironments shape how major hypoxia-responsive modules are engaged: 1) a HIF-1α-driven inflammatory transcriptional program (enhanced cytokine/chemokine output, leukocyte adhesion and recruitment signals, and prolonged activation/survival of innate immune cells); 2) a VEGF-centered vascular program (angiogenesis, increased permeability, edema, and remodeling); and 3) hypoxia-driven metabolic remodeling [greater reliance on glycolysis with lactate accumulation/acidification, altered mitochondrial redox balance, amplification of protease/reactive oxygen species (ROS) injury, and impaired tissue repair]. This shared-yet-context-dependent framing helps explain clinical heterogeneity and highlights hypoxia pathways as potentially cross-cutting therapeutic targets across respiratory inflammatory diseases.

## 3 Mechanisms underlying hypoxia-induced respiratory inflammation

### 3.1 Regulatory effect of hypoxia on airway epithelial cells

**Fig. 3:**
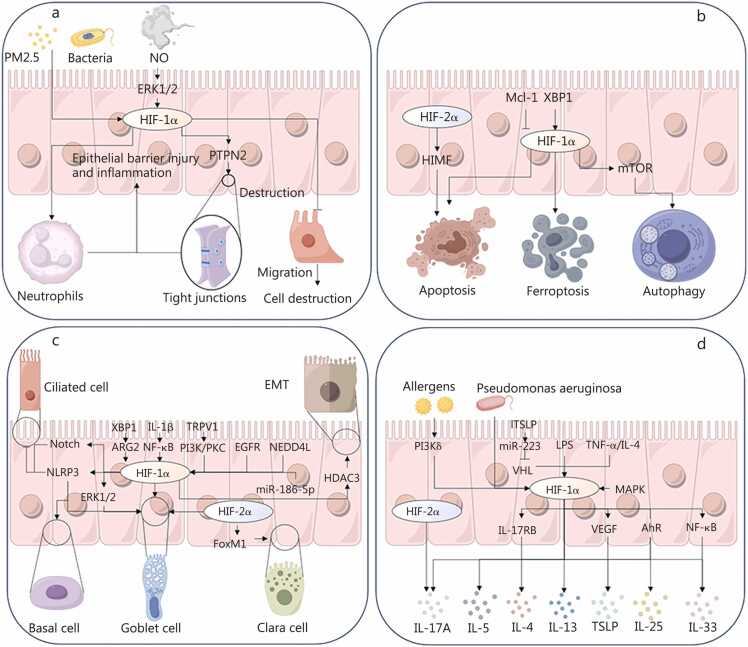
Role of hypoxia in airway epithelial cell pathology. Hypoxia disrupts barrier function and impairs ciliary-mucus clearance in respiratory epithelial cells (**a**), initiating a cascade of cellular stress responses. This includes induction of epithelial cell death, autophagy, and ferroptosis (**b**), as well as dysregulated cellular differentiation and EMT (**c**). Concurrently, hypoxia drives the secretion of inflammatory mediators and cytokines (**d**), which collectively exacerbate tissue damage and promote disease pathogenesis. These mechanisms highlight how hypoxic microenvironments systematically compromise epithelial homeostasis, linking cellular dysfunction to the development of inflammatory airway disorders. PM2.5. Particulate matter 2.5; NO. Nitric oxide; ERK1/2. Extracellular signal-regulated kinase 1/2; HIF-1α. Hypoxia-inducible factor-1α; PTPN2. Protein tyrosine phosphatase non-receptor type 2; Mcl-1. Myeloid cell leukemia-1; XBP1. X-box binding protein 1; HIMF. Hypoxia-inducible mitogenic factor; mTOR. Mammalian target of rapamycin; EMT. Epithelial-mesenchymal transition; IL. Interleukin; TRPV1. Transient receptor potential vanilloid 1; PI3K. Phosphatidylinositol 3-kinase; PKC. Protein kinase C; EGFR. Epidermal growth factor receptor; NEDD4L. Neural precursor cell expressed developmentally downregulated 4-like; HDAC3. Histone deacetylase 3; miR. MicroRNA; FoxM1. Forkhead box M1; IL-17RB. Interleukin-17 receptor B; VEGF. Vascular endothelial growth factor; AhR. Aryl hydrocarbon receptor; NF-κB. Nuclear factor κB; TSLP. Thymic stromal lymphopoietin; LPS. Lipopolysaccharide; TNF-α tumor necrosis factor-α; PI3Kδ. Phosphatidylinositol 3-kinase delta; MAPK. Mitogen-activated protein kinase; NLRP3 NOD-like receptor family pyrin domain containing 3; ARG2 anterior gradient 2; VHL von Hippel-Lindau; ITSLP. Long isoform thymic stromal lymphopoietin.

The airway epithelium, spanning the nasal cavity and conducting airways, serves as the first and most critical barrier against inhaled pollutants, pathogens, and allergens [73], [149]. Hypoxia can compromise this barrier through direct epithelial dysfunction and/or inflammation-driven injury, weakening junctional integrity and mucociliary defenses. Barrier disruption facilitates retention and accumulation of noxious stimuli within the airway lumen, which further amplifies epithelial activation and immune cell recruitment, thereby establishing a self-perpetuating inflammatory loop that progressively erodes barrier function [150], [151], [152] **(**Fig. 3**)**.

#### 3.1.1 Hypoxia-driven airway epithelial injury, barrier breakdown, and maladaptive remodeling

Hypoxia-driven epithelial injury compromises two core airway defense functions, immune regulation and ciliated mucus clearance, thereby initiating and perpetuating airway inflammation [78], [153], and hypoxia further exacerbates this disruption through several convergent mechanisms. Hypoxia-induced stabilisation of HIF-1α destabilises epithelial tight junctions, in part by inhibiting protein tyrosine phosphatase nonreceptor type 2 (PTPN2) and by inducing VEGF, leading to increased permeability and facilitating the ingress of inflammatory triggers [154], [155]. Consistent with these barrier-disrupting effects, elevated HIF-1α is also associated with bronchial epithelial damage [156]. Nitric oxide inhibits epithelial cell migration by suppressing extracellular signal-regulated kinase1/2 (ERK1/2) activity and promoting HIF-1α and p53 signalling, thereby contributing to cell injury [157]. HIF-1α also promotes bacterial flagellin-induced barrier dysfunction [158]. HIF-1α intensifies neutrophil degranulation and exacerbates epithelial damage under hypoxia [112]. Furthermore, beyond the mechanisms described above, Olson *et al*. [159] demonstrated that HIF-1 activation induces oxidative stress and impairs barrier integrity.

Beyond acute barrier failure, hypoxia also perturbs epithelial development and differentiation, promoting maladaptive remodeling that impairs mucociliary clearance and contributes to mucus plugging, airflow obstruction, and chronic inflammation [61], [62], [77], [78], [160], and HIFs, particularly HIF-1α and HIF-2α, function as master regulators in this process [78], [84], [161], [162]. Activated HIF-1α promotes basal cell proliferation while suppressing differentiation toward ciliated lineages [163]. In parallel, HIF-1α biases differentiation toward goblet cells, enhances mucin biosynthesis, and increases MUC5AC secretion [61], [164], [165], effects that may be linked to inflammation-triggered activation of downstream inflammatory pathways under inflammatory stimulation [160], [166], [167]. HIF-2α can also contribute to epithelial dysfunction by promoting Clara (club) cell proliferation via forkhead box M1 (FoxM1) signaling [168]. Moreover, hypoxia can induce epithelial-mesenchymal transition (EMT), a key process in epithelial dysfunction and airway remodeling, largely through hypoxia-driven stabilization and accumulation of HIF-1α and activation of downstream EMT programs [73], [77], [169], [170], [171]. Abnormalities in the CFTR gene in patients with CF disrupt chloride and sodium ion transport, leading to dehydration of the airway surface liquid (ASL) and impaired ciliary function [12], [106], [107], and hypoxia further exacerbates these defects [172], [173], [174], in part by increasing ROS production, which reduces ciliary beat frequency and compromises mucociliary transport [62].

#### 3.1.2 Inflammatory signaling activation and induction of cell death pathways

Persistent hypoxia disrupts epithelial barrier integrity, increasing epithelial susceptibility to inhaled irritants and pathogens and driving heightened release of proinflammatory mediators (e.g., lysozyme and interferons), which in turn promotes immune-cell recruitment and tissue infiltration [175], [176], [177], [178], [179].

Under hypoxic conditions, stabilized/activated HIF-1α accelerates intraepithelial inflammatory mediator release, thereby amplifying and propagating the inflammatory cascade [89], [180]. Activated HIF-1α subsequently induces the transcription of multiple epithelial-derived cytokines, notably interleukin (IL)-25, IL-33, and thymic stromal lymphopoietin (TSLP), thereby promoting type 2 immune polarisation [181], [182]. In parallel, HIF-1α and HIF-2α regulate IL-17A production, and the PI3K/HIF-1α axis upregulates IL-25 and IL-17RB expression, providing a mechanistic bridge between type 2 and neutrophilic inflammation [183], [184]. Metabolically, the long isoform thymic stromal lymphopoietin (ITSLP)/miR-223/VHL/HIF-1α signaling axis can reprogram epithelial cells toward glycolysis, supporting sustained cytokine production [185]. Importantly, tumor necrosis factor-α (TNF-α) and IL-4, as well as MAPK pathway activation, upregulate HIF-1α expression, creating a feed-forward inflammatory loop [186], [187]. Allergens and PI3Kδ activation upregulate HIF-1α-driven VEGF production, promoting angiogenesis and amplifying the release of inflammatory mediators [188]. In addition, HIF-1α can increase epithelial ROS generation and suppress nuclear factor erythroid 2‑related factor 2 (Nrf2)-dependent antioxidant responses, weakening redox defenses and exacerbating epithelial inflammation [183], [189].

Hypoxia exacerbates multiple forms of cell death, amplifies inflammatory responses, and accelerates epithelial cell senescence, thereby further compromising epithelial barrier integrity. X-box binding protein 1 (XBP1) can upregulate HIF-1α and β-catenin, promoting apoptosis [190], [191]. In addition, HIF-1α not only drives apoptosis but also induces ferroptosis [192], [193] and, via mammalian target of rapamycin (mTOR) signalling, stimulates autophagy [194]. HIF-2α modulates apoptosis via hypoxia-induced mitogenic factor (HIMF) [195], while myeloid cell leukemia (Mcl-1) may counteract cell death by regulating HIF-1α activity [196]. ROS accumulation under hypoxia amplifies death pathways [145]. Activation of the NOD-like receptor family pyrin domain-containing 3 (NLRP3) inflammasome by HIF-1α links epithelial cell death to inflammatory amplification [197], [198]. Emerging evidence also suggested that HIF-1α modulates epithelial ageing [199].

### 3.2 Regulatory effect of hypoxia on airway immune cells

**Fig. 4:**
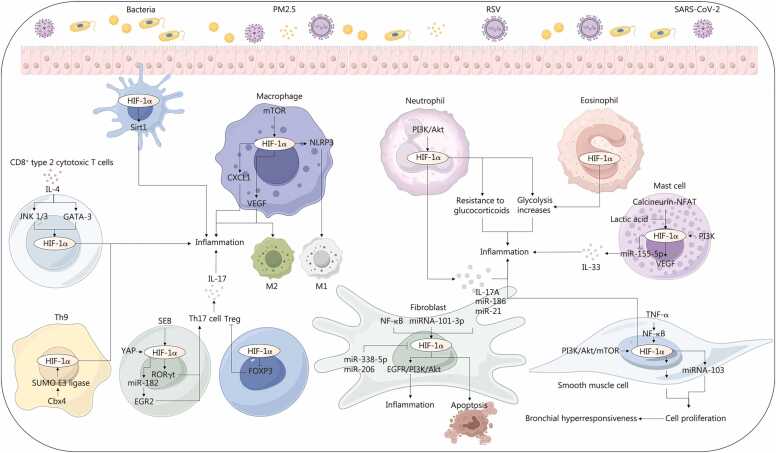
Hypoxia-mediated regulation of immune cell responses. Hypoxia in the respiratory tract drives immune dysfunction and inflammatory pathogenesis by orchestrating the activation of macrophages, eosinophils, neutrophils, mast cells, T cells, antigen-presenting cells, smooth muscle cells, and fibroblasts. This cascade sustains chronic immune activation and inflammatory states. HIF-1α. Hypoxia-inducible factor-1α; Sirt1. sirtuin 1; mTOR. Mammalian target of rapamycin; NLRP3. NOD-like receptor family pyrin domain containing 3; CXCL1. C-X-C motif chemokine ligand 1; VEGF. Vascular endothelial growth factor; PI3K/Akt. Phosphatidylinositol 3-kinase/protein kinase B; IL. Interleukin; JNK1/3. c-Jun N-terminal kinase 1/3; GATA-3. GATA binding protein 3; miR. MicroRNA; NF-κB. Nuclear factor κB; EGFR. Epidermal growth factor receptor; TNF-α. Tumor necrosis factor-α; MDM2. Murine double minute 2; p53. Tumor protein P53; SEB. Staphylococcal enterotoxin B; YAP. Yes-associated protein; RORγt. RAR-related orphan receptor γt; EGR2. Early growth response 2; FOXP3. Forkhead box P3; SUMO E3. Ligase small ubiquitin-like modifier E3 ligase; Cbx4. Chromobox homolog 4; PM2.5. Particulate matter 2.5; RSV. Respiratory syncytial virus; SARS-CoV-2. Severe acute respiratory syndrome coronavirus 2; NFAT. Nuclear factor of activated T-cells.

Persistent hypoxia further aggravates epithelial injury and dysfunction, thereby enhancing the recruitment and activation of resident and infiltrating immune cells, including macrophages [200], neutrophils [201], eosinophils [202], and other leukocytes, and amplifying the inflammatory cascade [203]. Activated immune cells then release additional cytokines, chemokines, proteases, and ROS, which feed back to worsen epithelial damage and barrier disruption [204], [205], [206], [207]. This establishes a self-perpetuating positive-feedback loop that sustains and escalates airway inflammation (Fig. 4).

#### 3.2.1 Immune modulation and pathological proliferation

HIF-1α is generally associated with proinflammatory M1 polarisation, whereas HIF-2α tends to promote anti-inflammatory M2 differentiation [24], [35]. However, HIF-1α has also been implicated in M2 macrophage polarization; for example, glutathione s-transferase omega 1 deficiency can paradoxically increase both HIF-1α expression and M2 polarisation [208], and HIF-1α/VEGF-A signalling may, under certain conditions, induce M2-like traits [209]. Staphylococcal enterotoxin B (SEB) stimulation increases HIF-1α expression, induces retinoic acid-related orphan receptor γt (RORγt) expression in Tregs, promotes a pathogenic phenotype [210], [211], and directly drives the expansion of Th17 cells with increased IL-17 production [212]. In addition, chromobox homologue 4 (CBX4) regulates HIF-1α through small ubiquitin-like modifier (SUMO) E3 ligase activity, promoting the differentiation of Th9 cells [213]. IL-4, via Janus kinase 1/3 (JAK1/3) and GATA binding protein 3 (GATA-3), increases HIF-1α-dependent differentiation of CD8^+^ type 2 cytotoxic T cells [98]. Interestingly, hypoxia inhibits adaptive immune responses by disrupting antigen-presenting cells differentiation, which is contrary to the functions of other immune cell types [214]. In addition, HIF-1α may also promote the proliferation of airway smooth muscle cells (ASMCs) via the miR-103 and murine double minute 2 (MDM2)-p53 axes [63], [215], whereas HIF-2α collaborates with HIF-1α to induce S100A4 expression, which may synergistically exacerbate the proliferation of airway vascular smooth muscle [216]. HIF-1α can act as a molecular link to drive fibroblast proliferation, promote differentiation into myofibroblasts, and increase extracellular matrix (ECM) production, ultimately promoting tissue remodeling [81], [217], [218], [219], [220], [221]. Previous studies suggested that HIF-1α can regulate fibroblast proliferation via non-coding RNAs [222], [223], [224], [225].

#### 3.2.2 Activation of inflammatory signaling

In macrophages, HIF-1α activation induces VEGF‑A and C-X-C motif chemokine ligand 1 (CXCL1) production, thereby promoting airway angiogenesis and inflammatory cell recruitment [226], [227], and it also acts in concert with MAPK and glutamate dehydrogenase (GDH) to regulate the generation of immunoregulatory mediators [228]. During airway inflammation, HIF-1α directly promotes Th17 expansion, increases IL-17 production, and sustains neutrophil‑dominant inflammation [212]. Consistently, neutrophil recruitment and activation are mediated in part by the PI3K/Akt/HIF-1 axis [229], which strengthens neutrophil-driven responses and has been associated with glucocorticoid resistance [230]. In airway smooth muscle, TNF-α increases *HIF-1α* mRNA synthesis via a nuclear factor κB (NF-κB)-dependent mechanism, thereby inducing a broader set of proinflammatory genes [64]. Additionally, PI3K/Akt/mTOR and ERK signaling activate HIF-1α and further reinforce the inflammatory state [231]. In mast cells, hypoxia upregulates HIF-1α transcription via the calcineurin-NFAT pathway, accelerating inflammatory progression [232], while activation of the PI3K/HIF-1α/VEGF axis increases vascular permeability, promoting airway edema and inflammatory infiltration [100]. Moreover, Fyn kinase and vesicle-associated membrane proteins (VAMPs) facilitate VEGF release from mast cells, further exacerbating respiratory tract inflammation [233], and mast cell-derived lactate suppresses miR-155-5p via HIF-1α, thereby modulating IL-33-triggered airway inflammation [234]. Finally, in respiratory tract dendritic cells, HIF-1α is regulated through a SIRT1-dependent mechanism and influences IgE, leukotriene C4 (LTC4), and eosinophil cationic protein (ECP) levels, collectively promoting airway inflammation [45].

#### 3.2.3 Altered metabolism

HIF-1α is also linked to enhanced glycolytic metabolism in neutrophils, eosinophils, and Th17 cells, which can promote immune imbalance and thereby exacerbate airway inflammation [99], [109], [111]. In macrophages, metabolic crosstalk mediated by enzymes and signaling nodes, including MAPK, HIF-1α, and glutamate dehydrogenase (GDH), can further amplify the production and release of inflammatory mediators, worsening airway inflammatory responses [228]. Additionally, lactate accumulation in airway mast cells and dysregulated lipid metabolism in Th17 cells may potentiate immune cell inflammatory mediator output through HIF-1α dependent regulation of non-coding RNAs, ultimately intensifying and sustaining the inflammatory milieu [234], [235].

## 4 Links between respiratory disease triggers and hypoxia

### 4.1 Viruses

HIF-1α activation by RSV viral infection increases glycolysis and viral replication [136]. Additionally, pharmacologic or genetic inhibition of HIF-1α and HIF-2α reduces RSV replication and pathogenesis [236], [237], [238]. Protein kinase C (PKC)δ/HIF-1α/NF-κB signaling regulates RSV replication and epithelial responses [239]. Similarly, in influenza virus infection, HIF-1α upregulation enhances viral replication and cytokine release through hexokinase 2 (HK2)-dependent glycolysis [71], [240], [241], [242], [243], [244], [245], [246].

**Fig. 5:**
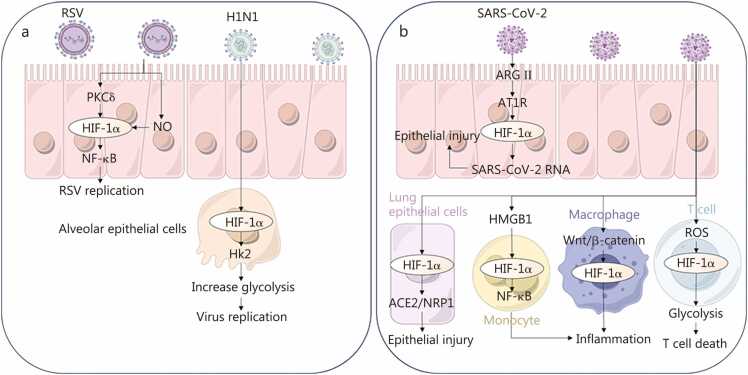
Role of hypoxia in respiratory viral infection-driven inflammation. **a** RSV exacerbates respiratory epithelial damage via hypoxia, whereas the influenza virus potentiates inflammatory progression in alveolar epithelial cells. **b** SARS-CoV-2 infection induces respiratory epithelial injury through hypoxic signaling pathways, triggering the activation of lung epithelial cells, T cells, monocytes, and macrophages. This cascade promotes cellular damage and sustains inflammatory states. RSV. Respiratory syncytial virus; H1N1. Influenza A virus subtype H1N1; PKCδ. Protein kinase C delta; HIF-1α. Hypoxia-inducible factor-1α; NO. Nitric oxide; NF-κB. Nuclear factor κB; Hk2. Hexokinase 2; SARS-CoV-2. Severe acute respiratory syndrome coronavirus 2; ARG II. Arginase II; AT1R. Angiotensin II type 1 receptor; HMGB1. High-mobility group box 1; ROS. Reactive oxygen species; ACE2. Angiotensin-converting enzyme 2; NRP1. Neuropilin 1; Wnt. Wingless-type MMTV integration site family.

SARS-CoV-2 infection engages a broader HIF-1α-centered regulatory network that reshapes host metabolism, inflammation, and tissue injury [141], [247], [248], [249], [250], [251], [252], [253]. SARS-CoV-2 can increase HIF-1α-dependent transcriptional programs that favor viral RNA and nucleocapsid protein production, contributing to epithelial damage and barrier dysfunction [254]. Mechanistically, the virus suppresses oxidative phosphorylation (OXPHOS) gene expression, induces miR-2392, and activates HIF-1α together, shifting cellular metabolism toward glycolysis and promoting immune activation [255]. Upstream, the accumulation of angiotensin II and the subsequent activation of the angiotensin II type 1 receptor (AT1R) facilitate the nuclear translocation of HIF-1α [256], whereas mitochondrial ROS and the viral open reading frame 3a (ORF3a) protein provide additional signals to increase HIF-1α stabilisation [140], [257]. Downstream, HIF-1α activation in monocytes drives NF-κB signaling, resulting in the production of proangiogenic and proinflammatory mediators [258], [259]. In the adaptive immune compartment, SARS-CoV-2 infection of T cells initiates a ROS-HIF-1α axis that culminates in T-cell death [138], [145]. Moreover, the viral non-structural protein 16 (NSP16) increases IL-6 expression in a HIF-1α-dependent manner [260], and Wingless-type MMTV integration site family (Wnt)/β-catenin signalling acts in concert to increase HIF-1α activity [261]. Under hypoxic conditions, several studies report angiotensin-converting enzyme 2 (ACE2) downregulation, which may confer resistance to viral entry by reducing the availability of the major SARS-CoV-2 receptor on the cell surface [144], [254], [262], [263], [264], [265], [266]. However, in established infection or prolonged hypoxia, alternative regulatory programs may emerge; notably, HIF-1α has also been reported to induce ACE2 expression in certain settings [267] **(**Fig. 5**)**.

### 4.2 Bacteria

Acute and recurrent bacterial infections are closely linked to hypoxia-driven airway inflammation, as they promote neutrophil recruitment/aggregation and are accompanied by increased HIF-1α expression [52]. In bronchial epithelial cells, HIF-1α can further aggravate flagellin-associated disruption of epithelial barrier integrity [158]. Beyond inflammatory cell infiltration, microbial products such as lipopolysaccharide (LPS) promote the stabilization and accumulation of HIF-1α and HIF-2α via multiple signaling pathways, including PI3K-dependent routes, thereby amplifying airway inflammation [183], [268]. In CF airway epithelial cells, *Pseudomonas aeruginosa* infection has been reported to increase HIF-1α protein abundance [109]. Additionally, *Poraulatracochlax* infection may exacerbate inflammation by activating HIF-1α signaling through NF-κB [269].

### 4.3 Allergens

Allergen exposure can directly upregulate HIF-1α and VEGF expression in patients with asthma and AR, thereby promoting allergic airway inflammation [41], [188]. Sensitizing fungi (e.g., *Alternaria*) can further enhance airway HIF-1α expression and trigger inflammatory mediator release, reinforcing allergic inflammatory cascades [52].

### 4.4 Pollutants

Smoking promotes inflammation in COPD via the specific protein 1 (Sp1)/SIRT1/HIF-1α pathway [199] and may further exacerbate COPD inflammation by inhibiting ferroptosis in alveolar epithelial cells through the HIF-3α-GPX4 axis [131]. Moreover, long-term cigarette exposure can aggravate eosinophilic airway inflammation by increasing HIF-1α and VEGF expression, thereby promoting airway remodeling and nasal polyp formation [270]. Nickel nanoparticle exposure increases HIF-1α-driven matrix metalloproteinase (MMP)-2 and MMP-9 production, worsening allergic airway inflammation [53]. In addition, PM2.5 enhances Th17-cell glycolysis through HIF-1α and AhR signaling to intensify airway inflammation [99]. Nitric oxide can aggravate inflammatory airway injury by inhibiting ERK1/2 signaling and promoting activation of HIF-1α and p53 [157]. Collectively, these findings suggest that environmental toxicants can aggravate HIF-associated pathological changes.

## 5 Targeting HIF-1α and associated pathways

Given that HIF-1α functions as a key pathogenic driver in several respiratory diseases, the development of therapeutic strategies that target HIF-1α and its downstream signaling pathways is of critical importance. A growing number of interventions, including glucocorticoids and oxygen therapy, small molecule compounds, plant extracts, and traditional Chinese medicines (TCMs), have been shown to markedly attenuate HIF-1α-mediated pathogenic effects. By regulating HIF-1α-mediated angiogenic signals [89], [209], [271], [272], [273], [274], [275], [276], [277], [278], [279], inflammatory pathways [58], [280], [281], [282], [283], [284], [285], [286], [287], [288], [289], [290], [291], [292], [293], [294], metabolic processes [295], and cell fate decisions [190], [290], these interventions can counteract hypoxia-related pathological changes in the airways. Among available options, glucocorticoids and oxygen therapy are the most clinically mature and provide direct mechanistic support for the hypoxia/HIF axis as a therapeutic target [296], [297]. Glucocorticoids can downregulate HIF-1α and its downstream target VEGF, thereby reducing airway hyperresponsiveness in experimental models [296]. Meanwhile, they can also modulate the HIF-1α-mediated glycolysis-lactic acid axis to restrict the development of eosinophilic inflammation [296], [298]. Oxygen therapy, a first-line treatment for hypoxemia in diseases such as COPD, improves symptoms (e.g., dyspnea) and can reduce the risk of acute exacerbations in clinical studies [299], [300]. Mechanistically, increasing local oxygen tension promotes HIF-1α ubiquitination and proteasomal degradation, suppressing downstream proinflammatory and pro-remodeling factors [29], [32], [34]. Together, these observations reinforce HIF signaling as a central node in disease progression and a rational therapeutic target.

During the preclinical stage, selective HIF inhibitors and metabolic regulators show strong translational potential. However, their development should follow a staged pathway progressing from rigorous mechanistic validation, to target and lead optimization, and ultimately to clinically oriented translation. For selective HIF inhibition, YC-1 (a benzylindazole derivative) suppresses HIF-1α activity and also intersects with NF-κB and PPAR-γ signaling. In animal models of asthma and AR, YC-1 reduces airway responsiveness and Th2 cytokines (e.g., IL-4, IL-5). Next steps would include optimizing pharmacokinetics (e.g., improving lung exposure/retention) and then conducting Phase I/II trials in moderate-to-severe asthma to establish safety and preliminary efficacy in humans [278], [301], [302]. Metabolic modulators such as adenosine monophosphate-activated protein kinase (AMPK) activators can alleviate airway inflammation by inhibiting the expression of HIF-1α and HIF-2α and reducing the production of ROS. Given the core role of AMPK in energy metabolism, clinical exploration can be prioritized in COPD patients with comorbid metabolic abnormalities (e.g., obesity) to achieve precise stratified treatment based on patients’ metabolic characteristics [303]. Additionally, roxadustat (FG-4592), a prolyl hydroxylase inhibitor originally developed for renal anemia, has been reported in preclinical work to inhibit SARS-CoV-2 replication, reduce epithelial injury, and improve mucociliary clearance, potentially via reducing HIF-1α expression [254]. This positions roxadustat as a candidate for viral respiratory infections (e.g., COVID-19, influenza), but its antiviral mechanism should be validated in human bronchial epithelial cell systems and followed by small, carefully monitored clinical pilot studies evaluating short-term outcomes in infected patients.

Many TCMs have been predicted (computationally) to regulate the HIF-1α pathway [304], [305], [306], [307], [308], [309], [310], [311], [312], [313], [314], [315], [316]. For instance, *Meliae cortex* and FangYi XiangNang have been proposed to act on the HIF-1α axis based on in silico approaches [317], [318]; reproducible *in vitro* evidence in human airway-relevant systems remains limited. Current support largely comes from preliminary or observational findings suggesting possible benefit through HIF-1α-related mechanisms, whereas high-quality clinical evidence is lacking [273], [274], [279], [283].

## 6 Integrative perspective and future directions

Clinical and experimental evidence suggests that HIF-1α is a central hub in respiratory inflammatory diseases, linking epithelial barrier dysfunction, immune dysregulation, and tissue remodeling. In COPD and asthma, hypoxia stabilizes HIF-1α, alters tight junction proteins, boosts inflammatory cytokines from macrophages and neutrophils, and promotes fibroblast proliferation and collagen deposition, accelerating remodeling [81], [319]. Thus, HIF-1α is a promising therapeutic target, best approached with precision strategies.

From a clinical translation perspective, the most productive avenues for future HIF-1α-targeted research fall into four areas. First, strengthen clinical translational research on the HIF-1α-VEGF axis by validating its biomarker and therapeutic relevance in well-designed patient cohorts and prospective studies. As a key downstream effector of HIF-1α, VEGF not only drives pathological angiogenesis in respiratory disease but also worsens airway edema by increasing vascular permeability. Anti-VEGF agents have demonstrated clinical benefit in oncology trials, suggesting that this axis could be further refined as a relatively mature intervention node for airway inflammation [320], [321], [322]. Second, develop selective HIF-1α modulators with improved target specificity and safety profiles to enable precise therapeutic intervention. Most current approaches rely on repurposed, non-specific agents, whereas small molecules that directly and selectively modulate HIF-1α activity remain largely preclinical [254], [278], [296], [301], [302]. Greater selectivity could help preserve physiological hypoxia adaptation in healthy tissues and reduce treatment-related risk. Third, optimize local delivery strategies to enhance tissue targeting and drug retention while minimizing systemic exposure and off-target toxicity. Given the potential off-target effects of systemic HIF inhibition, aerosolized formulations and intratracheal local gel delivery may enable preferential drug accumulation in lung tissue. For example, an aerosolized HIF-1α siRNA formulation validated in animal models markedly reduced pulmonary HIF-1α expression without overt systemic toxicity, highlighting an approach that could substantially improve the therapeutic index [188]. Fourth, accelerate biomarker development to enable robust patient stratification and guide personalized therapy. Targeted therapies are unlikely to benefit all patients uniformly. For instance, COPD trials of oxygen therapy indicated that only a subset of patients derive benefit from long-term oxygen therapy [299], [300]. Serum levels of HIF-1α downstream target genes may therefore serve as predictive biomarkers to identify patients most likely to respond to HIF-targeted interventions and to support individualized treatment.

## 7 Conclusions

In respiratory inflammatory diseases, hypoxia/HIF-1α signaling can disrupt epithelial barrier integrity, reprogram innate and adaptive immune responses, and promote vascular and tissue remodeling, collectively establishing a self-reinforcing “hypoxia-inflammation-structural damage” vicious cycle. This framework helps explain both shared pathogenic mechanisms and disease-specific phenotypic heterogeneity. Future translational work should prioritize HIF-1α-centered strategies, including biomarker-guided patient stratification, optimized local delivery, and selective pathway modulation, to rigorously evaluate efficacy and safety and ultimately enable precision therapy.

## Abbreviations

CBP: CREB-binding protein

HIF: Hypoxia-inducible factor

VHL: Von Hippel-Lindau

VEGF: Vascular endothelial growth factor

PI3K: Phosphoinositide 3-kinase

Akt: Protein kinase B

AhR: Aryl hydrocarbon receptor

PPAR-γ: Peroxisome proliferator-activated receptor-γ

PKC: Protein kinase C

CF: Cystic fibrosis

CRS: Chronic rhinosinusitis

AR: Allergic rhinitis

COPD: Chronic obstructive pulmonary disease

ERK1/2: Extracellular signal-regulated kinase1/2

CFTR: Cystic fibrosis transmembrane conductance regulator

ROS: Reactive oxygen species

NF-κB: Nuclear factor κB

TNF-α: Tumor necrosis factor-α

MAPK: Mitogen-activated protein kinase

mTOR: Mammalian target of rapamycin

SIRT1: Sirtuin1

FoxM1: Forkhead box M1

SARS-CoV-2: Severe acute respiratory syndrome coronavirus 2

Wnt: Wingless-type MMTV integration site family

IL: Interleukin

RSV: Respiratory syncytial virus

ITSLP: Long isoformthymic stromal lymphopoietin

Sp1: Specific protein 1

## Ethics approval and consent to participate

Not applicable.

## Funding

This work was supported by the National Natural Science Foundation of China (82401328 to BZ, 82403113 to SLS) and the China Postdoctoral Science Foundation (2024M762252).

## Data Availability

Not applicable.
